# 4-[2-(1-Acetyl-2-oxopropyl­idene)­hydrazino]-*N*-(pyrimidin-2-yl)benzene­sulfonamide

**DOI:** 10.1107/S1600536809018765

**Published:** 2009-05-23

**Authors:** Priyanka Rai, Shalini Upadhyay, M. Nethaji, K. K. Upadhyay

**Affiliations:** a Department of Chemistry, Udai Pratap College (Autonomous), Varanasi 221 002, India; b Department of Chemistry, Faculty of Science, Banaras Hindu University, Varanasi 221 005, India; c Department of Inorganic and Physical Chemistry, Indian Institute of Sciences, Bangalore 560 012, India

## Abstract

In the title compound, C_15_H_15_N_5_O_4_S, the dihedral angle between the pyrimidine and benzene rings is 84.56 (2)°. Intra­molecular hydrazine–carbonyl N—H⋯O and inter­molecular sulfonamide–pyridimine N—H⋯N hydrogen bonds stabilize the mol­ecular and crystal structures, respectively.

## Related literature

For background to sulfa drugs and their derivatives, see: Abbate *et al.* (2004 ▶); Badr (2008 ▶); Gale *et al.* (2007 ▶); Hanafy *et al.* (2007 ▶); Novinson *et al.* (1976 ▶); Supuran *et al.* (2003 ▶). For the synthesis of the title compound, see: Goyal & Bhargava (1989 ▶).
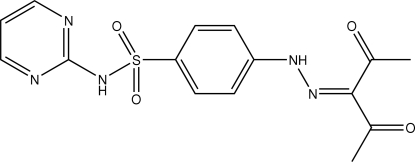


## Experimental

### 

#### Crystal data

C_15_H_15_N_5_O_4_S
*M* *_r_* = 361.38
Monoclinic, 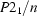

*a* = 11.354 (3) Å
*b* = 5.7875 (13) Å
*c* = 25.974 (6) Å
β = 101.877 (4)°
*V* = 1670.3 (7) Å^3^
*Z* = 4
Mo *K*α radiation
μ = 0.23 mm^−1^
*T* = 293 K
0.24 × 0.22 × 0.20 mm

#### Data collection

Bruker SMART APEX diffractometer
Absorption correction: empirical (using intensity measurements) (*SADABS*; Bruker, 2005 ▶) *T*_min_ = 0.947, *T*_max_ = 0.957
17935 measured reflections
3999 independent reflections
3164 reflections with *I* > 2σ(*I*)
*R*_int_ = 0.027

#### Refinement

*R*[*F* ^2^ > 2σ(*F* ^2^)] = 0.047
*wR*(*F* ^2^) = 0.135
*S* = 1.07
3999 reflections
228 parameters
H-atom parameters constrained
Δρ_max_ = 0.43 e Å^−3^
Δρ_min_ = −0.27 e Å^−3^

### 

Data collection: *APEX2* (Bruker, 2008 ▶); cell refinement: *SAINT* (Bruker, 2008 ▶); data reduction: *SAINT*; program(s) used to solve structure: *SHELXS97* (Sheldrick, 2008 ▶); program(s) used to refine structure: *SHELXL97* (Sheldrick, 2008 ▶); molecular graphics: *ORTEP-3 for Windows* (Farrugia, 1997 ▶); software used to prepare material for publication: *WinGX* (Farrugia, 1999 ▶).

## Supplementary Material

Crystal structure: contains datablocks I, global. DOI: 10.1107/S1600536809018765/tk2450sup1.cif

Structure factors: contains datablocks I. DOI: 10.1107/S1600536809018765/tk2450Isup2.hkl

Additional supplementary materials:  crystallographic information; 3D view; checkCIF report

## Figures and Tables

**Table 1:** Hydrogen-bond geometry (Å, °)

*D*—H⋯*A*	*D*—H	H⋯*A*	*D*⋯*A*	*D*—H⋯*A*
N2—H2*A*⋯O3	0.86	2.01	2.654 (2)	131
C15—H15⋯O4^i^	0.93	2.46	3.262 (3)	144

Symmetry code: (i) 
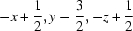
.

**Fig. 1.**
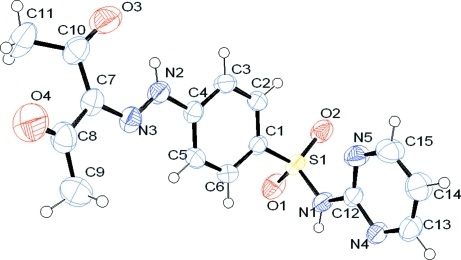
The molecular structure of (I) showing the atom-labeling scheme. Displacement ellipsoids are drawn at the 50% probability level.

**Fig. 2.**
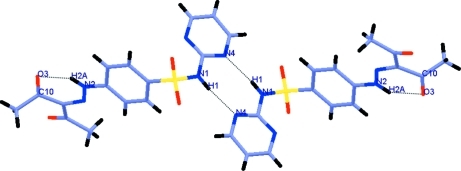
View of (I) showing intermolecular and intramolecular hydrogen bonding, as thin lines.

## supplementary crystallographic information

### Comment

Sulfa drugs and their derivatives have attracted much attention due to their wide spectrum of applications as compounds with anti-bacterial (Badr, 2008), anti-fungal (Hanafy *et al.*, 2007), anti-viral (Supuran *et al.*, 2003), anti-malarial (Gale *et al.*, 2007), and anti-cancer (Abbate *et al.*, 2004) activities. Although the title compound (I) been reported in the literature (Goyal & Bhargava, 1989), its crystal structure was not reported. The molecule of (I), Fig. 1, is-non planar as seen in arrangement of the two aromatic moieties attached to sulfonamide, –NHSO_2_-, group; the C1—S1—N1—C12 torsion angle is 68.66 (15)°. Within the molecule, there is a prominent intramolecular interaction between the hydrazo-N—H and the carbonyl-O3 atoms, Fig. 2 and Table 1. Intermolecular hydrogen bonds formed between centrosymmetrically related molecules involving the sulfonamide-N—H and the pyrimidine-N4 atoms lead to dimeric aggregates, Fig. 2 and Table 1.

### Experimental

Compound (I) was synthesized using the literature procedure (Novinson *et al.*, 1976) as follows. Sulfadiazine (2 mmol, 501 mg) and sodium nitrite (~4 mmol, 300 mg) were dissolved separately in conc. HCl (2 ml) and distilled water (10 ml), respectively, followed by their cooling on crushed ice. The cooled sodium nitrite solution was added to the sulfdiazine solution with constant stirring while maintaining ice-cold temperature. The resulting yellow solution was added to a mixture of acetyl acetone (2 mmol, 0.2 ml) and sodium acetate (~37 mmol, 3 g) in distilled water (15 ml) with continuous stirring. The stirring was continued for 2 h maintaining the temperature of the reaction vessel between 293–298 K. The resulting solids were filtered, washed with water, ethanol and finally, by diethyl ether. The crude product was recrystallized from a water–ethanol mixture (50% *v*/*v*) and dried *in vacuo*. Crystals of (I) were developed by layering its supersaturated solution in ethanol with diethylether and leaving for a few days.

Yield 78%. Spectroscopic anaylysis: ^1^H NMR (DMSO-d_6_, TMS, δ p.p.m.) 13.51 (1H, NH) 11.79 (1H, NH), 8.51–7.04 (7H, Ar—H), 2.54–2.42 (6H, CH_3_). ^13^C NMR (DMSO-d_6_, TMS, δ p.p.m.) 197.41, 196.40 (>C═O), 158.3, 156.8, 145.44 (>C═C<), 135.4, 135.3, 129.35, 115.73 (ArC), 31.18, 26.24 (CH_3_).

### Refinement

All H atoms were placed in the idealized positions with C—H = 0.93–0.96 Å and N—H = 0.86 Å, and with *U*_iso_(H) = 1.2—1.5 *U*_eq_(C, N).

### Crystal data

**Table:**

C_15_H_15_N_5_O_4_S	*F*(000) = 752
*M**_r_* = 361.38	*D*_x_ = 1.437 Mg m^−^^3^
Monoclinic, *P*2_1_/*n*	Melting point: 508 K
Hall symbol: -P 2yn	Mo *K*α radiation, λ = 0.71073 Å
*a* = 11.354 (3) Å	Cell parameters from 489 reflections
*b* = 5.7875 (13) Å	θ = 2.5–27.5°
*c* = 25.974 (6) Å	µ = 0.23 mm^−^^1^
β = 101.877 (4)°	*T* = 293 K
*V* = 1670.3 (7) Å^3^	Block, colourless
*Z* = 4	0.24 × 0.22 × 0.20 mm

### Data collection

**Table:**

Bruker SMART APEX diffractometer	3999 independent reflections
Radiation source: fine-focus sealed tube	3164 reflections with *I* > 2σ(*I*)
graphite	*R*_int_ = 0.027
Detector resolution: 0.3 pixels mm^-1^	θ_max_ = 28.2°, θ_min_ = 1.6°
ω scans	*h* = −14→14
Absorption correction: empirical (using intensity measurements) (*SADABS*; Bruker, 2005)	*k* = −7→7
*T*_min_ = 0.947, *T*_max_ = 0.957	*l* = −33→34
17935 measured reflections	

### Refinement

**Table:**

Refinement on *F*^2^	Primary atom site location: structure-invariant direct methods
Least-squares matrix: full with fixed elements per cycle	Secondary atom site location: difference Fourier map
*R*[*F*^2^ > 2σ(*F*^2^)] = 0.047	Hydrogen site location: inferred from neighbouring sites
*wR*(*F*^2^) = 0.135	H-atom parameters constrained
*S* = 1.07	*w* = 1/[σ^2^(*F*_o_^2^) + (0.0735*P*)^2^ + 0.3686*P*] where *P* = (*F*_o_^2^ + 2*F*_c_^2^)/3
3999 reflections	(Δ/σ)_max_ < 0.001
228 parameters	Δρ_max_ = 0.43 e Å^−^^3^
0 restraints	Δρ_min_ = −0.27 e Å^−^^3^

### Special details

**Table:**

Geometry. All e.s.d.'s (except the e.s.d. in the dihedral angle between two l.s. planes) are estimated using the full covariance matrix. The cell e.s.d.'s are taken into account individually in the estimation of e.s.d.'s in distances, angles and torsion angles; correlations between e.s.d.'s in cell parameters are only used when they are defined by crystal symmetry. An approximate (isotropic) treatment of cell e.s.d.'s is used for estimating e.s.d.'s involving l.s. planes.
Refinement. Refinement of *F*^2^ against ALL reflections. The weighted *R*-factor *wR* and goodness of fit *S* are based on *F*^2^, conventional *R*-factors *R* are based on *F*, with *F* set to zero for negative *F*^2^. The threshold expression of *F*^2^ > σ(*F*^2^) is used only for calculating *R*-factors(gt) *etc*. and is not relevant to the choice of reflections for refinement. *R*-factors based on *F*^2^ are statistically about twice as large as those based on *F*, and *R*- factors based on ALL data will be even larger.

### Fractional atomic coordinates and isotropic or equivalent isotropic displacement parameters (Å^2^)

**Table:**

	*x*	*y*	*z*	*U*_iso_*/*U*_eq_	
C1	0.63840 (13)	0.4007 (3)	0.17280 (6)	0.0382 (3)	
C2	0.65017 (14)	0.2375 (3)	0.21231 (6)	0.0423 (4)	
H2	0.6861	0.0958	0.2085	0.051*	
C3	0.60841 (14)	0.2856 (3)	0.25745 (6)	0.0442 (4)	
H3	0.6172	0.1775	0.2845	0.053*	
C4	0.55332 (13)	0.4960 (3)	0.26228 (6)	0.0403 (3)	
C5	0.54124 (15)	0.6595 (3)	0.22244 (7)	0.0441 (4)	
H5	0.5036	0.7998	0.2258	0.053*	
C6	0.58527 (14)	0.6128 (3)	0.17784 (6)	0.0435 (4)	
H6	0.5793	0.7229	0.1513	0.052*	
C7	0.41088 (16)	0.7698 (3)	0.35557 (7)	0.0508 (4)	
C8	0.32001 (18)	0.9556 (4)	0.35144 (8)	0.0640 (5)	
C9	0.2914 (2)	1.0962 (4)	0.30216 (10)	0.0776 (6)	
H9A	0.2278	1.0230	0.2775	0.116*	
H9B	0.3617	1.1078	0.2871	0.116*	
H9C	0.2663	1.2480	0.3102	0.116*	
C10	0.46123 (17)	0.6545 (4)	0.40679 (7)	0.0588 (5)	
C11	0.4767 (3)	0.7965 (5)	0.45552 (9)	0.0890 (8)	
H11A	0.5122	0.7037	0.4853	0.134*	
H11B	0.3996	0.8519	0.4599	0.134*	
H11C	0.5282	0.9255	0.4528	0.134*	
C12	0.48295 (14)	0.2255 (3)	0.05575 (6)	0.0410 (3)	
C13	0.31325 (16)	0.1234 (3)	−0.00211 (7)	0.0558 (5)	
H13	0.2584	0.1438	−0.0337	0.067*	
C14	0.29513 (18)	−0.0519 (4)	0.03058 (9)	0.0672 (5)	
H14	0.2297	−0.1513	0.0218	0.081*	
C15	0.37720 (18)	−0.0752 (4)	0.07679 (9)	0.0645 (5)	
H15	0.3662	−0.1929	0.0997	0.077*	
N1	0.58083 (12)	0.3715 (2)	0.06677 (5)	0.0448 (3)	
H1	0.5816	0.4870	0.0460	0.054*	
N2	0.51240 (12)	0.5382 (3)	0.30859 (5)	0.0473 (3)	
H2A	0.5336	0.4483	0.3353	0.057*	
N3	0.44158 (12)	0.7152 (3)	0.31136 (5)	0.0474 (3)	
N4	0.40737 (12)	0.2669 (2)	0.00980 (5)	0.0447 (3)	
N5	0.47232 (14)	0.0633 (3)	0.09050 (6)	0.0543 (4)	
O1	0.77449 (10)	0.5273 (3)	0.10851 (5)	0.0599 (3)	
O2	0.74016 (12)	0.1133 (2)	0.11958 (5)	0.0593 (3)	
O3	0.49670 (15)	0.4579 (3)	0.40757 (6)	0.0857 (5)	
O4	0.26722 (17)	0.9867 (4)	0.38702 (7)	0.1059 (6)	
S1	0.69587 (3)	0.34360 (8)	0.116167 (15)	0.04382 (10)	

### Atomic displacement parameters (Å^2^)

**Table:**

	*U*^11^	*U*^22^	*U*^33^	*U*^12^	*U*^13^	*U*^23^
C1	0.0377 (7)	0.0417 (8)	0.0330 (7)	0.0005 (6)	0.0023 (6)	−0.0020 (6)
C2	0.0458 (8)	0.0380 (8)	0.0418 (8)	0.0078 (6)	0.0060 (7)	−0.0003 (6)
C3	0.0475 (8)	0.0447 (8)	0.0393 (8)	0.0065 (7)	0.0064 (7)	0.0082 (6)
C4	0.0375 (7)	0.0457 (8)	0.0361 (7)	−0.0001 (6)	0.0037 (6)	−0.0044 (6)
C5	0.0471 (8)	0.0370 (8)	0.0473 (9)	0.0071 (7)	0.0076 (7)	−0.0012 (7)
C6	0.0494 (8)	0.0409 (8)	0.0381 (8)	0.0041 (7)	0.0043 (7)	0.0051 (6)
C7	0.0483 (8)	0.0587 (10)	0.0456 (9)	0.0016 (8)	0.0098 (7)	−0.0085 (8)
C8	0.0610 (11)	0.0676 (12)	0.0638 (12)	0.0124 (10)	0.0139 (9)	−0.0124 (10)
C9	0.0716 (13)	0.0735 (14)	0.0858 (16)	0.0217 (11)	0.0114 (12)	0.0035 (12)
C10	0.0551 (10)	0.0769 (13)	0.0471 (10)	0.0054 (9)	0.0167 (8)	−0.0013 (9)
C11	0.0990 (17)	0.118 (2)	0.0458 (11)	0.0011 (16)	0.0061 (11)	−0.0156 (12)
C12	0.0447 (8)	0.0441 (8)	0.0336 (7)	−0.0011 (7)	0.0066 (6)	−0.0013 (6)
C13	0.0510 (9)	0.0650 (11)	0.0470 (9)	−0.0096 (9)	−0.0002 (7)	−0.0036 (8)
C14	0.0586 (11)	0.0666 (12)	0.0724 (13)	−0.0220 (10)	0.0041 (9)	0.0041 (10)
C15	0.0647 (11)	0.0609 (11)	0.0665 (12)	−0.0138 (10)	0.0104 (10)	0.0163 (10)
N1	0.0479 (7)	0.0494 (8)	0.0336 (6)	−0.0079 (6)	−0.0002 (5)	0.0049 (6)
N2	0.0508 (7)	0.0542 (8)	0.0367 (7)	0.0081 (6)	0.0085 (6)	0.0001 (6)
N3	0.0422 (7)	0.0553 (8)	0.0431 (7)	0.0034 (6)	0.0052 (6)	−0.0083 (6)
N4	0.0469 (7)	0.0509 (8)	0.0343 (6)	−0.0053 (6)	0.0038 (5)	−0.0001 (6)
N5	0.0582 (8)	0.0566 (9)	0.0457 (8)	−0.0083 (7)	0.0054 (6)	0.0123 (7)
O1	0.0471 (6)	0.0832 (9)	0.0491 (7)	−0.0162 (6)	0.0089 (5)	−0.0029 (6)
O2	0.0637 (7)	0.0686 (8)	0.0433 (6)	0.0224 (7)	0.0057 (5)	−0.0072 (6)
O3	0.1122 (12)	0.0941 (11)	0.0564 (8)	0.0341 (10)	0.0303 (8)	0.0152 (8)
O4	0.1151 (12)	0.1252 (15)	0.0895 (12)	0.0543 (12)	0.0496 (10)	−0.0011 (11)
S1	0.04035 (19)	0.0554 (2)	0.03392 (18)	0.00152 (17)	0.00343 (15)	−0.00326 (16)

### Geometric parameters (Å, °)

**Table:**

C1—C2	1.380 (2)	C10—C11	1.489 (3)
C1—C6	1.386 (2)	C11—H11A	0.9600
C1—S1	1.7590 (15)	C11—H11B	0.9600
C2—C3	1.381 (2)	C11—H11C	0.9600
C2—H2	0.9300	C12—N5	1.325 (2)
C3—C4	1.386 (2)	C12—N4	1.340 (2)
C3—H3	0.9300	C12—N1	1.378 (2)
C4—C5	1.388 (2)	C13—N4	1.339 (2)
C4—N2	1.397 (2)	C13—C14	1.366 (3)
C5—C6	1.380 (2)	C13—H13	0.9300
C5—H5	0.9300	C14—C15	1.366 (3)
C6—H6	0.9300	C14—H14	0.9300
C7—N3	1.306 (2)	C15—N5	1.333 (2)
C7—C8	1.478 (3)	C15—H15	0.9300
C7—C10	1.493 (3)	N1—S1	1.6396 (13)
C8—O4	1.214 (2)	N1—H1	0.8600
C8—C9	1.495 (3)	N2—N3	1.3136 (19)
C9—H9A	0.9600	N2—H2A	0.8600
C9—H9B	0.9600	O1—S1	1.4284 (13)
C9—H9C	0.9600	O2—S1	1.4210 (13)
C10—O3	1.206 (2)		
			
C2—C1—C6	120.88 (14)	C10—C11—H11A	109.5
C2—C1—S1	119.82 (12)	C10—C11—H11B	109.5
C6—C1—S1	119.27 (12)	H11A—C11—H11B	109.5
C1—C2—C3	119.71 (15)	C10—C11—H11C	109.5
C1—C2—H2	120.1	H11A—C11—H11C	109.5
C3—C2—H2	120.1	H11B—C11—H11C	109.5
C2—C3—C4	119.62 (15)	N5—C12—N4	127.00 (15)
C2—C3—H3	120.2	N5—C12—N1	118.39 (14)
C4—C3—H3	120.2	N4—C12—N1	114.61 (14)
C3—C4—C5	120.56 (15)	N4—C13—C14	122.19 (17)
C3—C4—N2	117.87 (14)	N4—C13—H13	118.9
C5—C4—N2	121.56 (14)	C14—C13—H13	118.9
C6—C5—C4	119.70 (15)	C13—C14—C15	117.17 (18)
C6—C5—H5	120.2	C13—C14—H14	121.4
C4—C5—H5	120.2	C15—C14—H14	121.4
C5—C6—C1	119.51 (15)	N5—C15—C14	122.95 (18)
C5—C6—H6	120.2	N5—C15—H15	118.5
C1—C6—H6	120.2	C14—C15—H15	118.5
N3—C7—C8	114.89 (17)	C12—N1—S1	125.49 (11)
N3—C7—C10	123.56 (16)	C12—N1—H1	117.3
C8—C7—C10	121.55 (16)	S1—N1—H1	117.3
O4—C8—C7	119.93 (18)	N3—N2—C4	119.95 (14)
O4—C8—C9	121.11 (18)	N3—N2—H2A	120.0
C7—C8—C9	118.91 (18)	C4—N2—H2A	120.0
C8—C9—H9A	109.5	C7—N3—N2	120.95 (15)
C8—C9—H9B	109.5	C13—N4—C12	115.42 (15)
H9A—C9—H9B	109.5	C12—N5—C15	115.25 (16)
C8—C9—H9C	109.5	O2—S1—O1	118.93 (9)
H9A—C9—H9C	109.5	O2—S1—N1	110.86 (6)
H9B—C9—H9C	109.5	O1—S1—N1	103.75 (6)
O3—C10—C11	121.73 (19)	O2—S1—C1	108.15 (6)
O3—C10—C7	120.14 (16)	O1—S1—C1	109.07 (6)
C11—C10—C7	117.9 (2)	N1—S1—C1	105.24 (7)
			
C6—C1—C2—C3	0.1 (2)	N4—C12—N1—S1	−172.23 (12)
S1—C1—C2—C3	177.95 (12)	C3—C4—N2—N3	−168.07 (14)
C1—C2—C3—C4	1.0 (2)	C5—C4—N2—N3	12.8 (2)
C2—C3—C4—C5	−0.8 (2)	C8—C7—N3—N2	−173.28 (15)
C2—C3—C4—N2	−179.93 (14)	C10—C7—N3—N2	6.1 (3)
C3—C4—C5—C6	−0.5 (2)	C4—N2—N3—C7	−173.92 (15)
N2—C4—C5—C6	178.57 (14)	C14—C13—N4—C12	0.5 (3)
C4—C5—C6—C1	1.6 (2)	N5—C12—N4—C13	−1.8 (3)
C2—C1—C6—C5	−1.4 (2)	N1—C12—N4—C13	178.52 (15)
S1—C1—C6—C5	−179.29 (12)	N4—C12—N5—C15	1.9 (3)
N3—C7—C8—O4	164.74 (17)	N1—C12—N5—C15	−178.47 (16)
C10—C7—C8—O4	−14.6 (3)	C14—C15—N5—C12	−0.6 (3)
N3—C7—C8—C9	−12.8 (3)	C12—N1—S1—O2	48.04 (14)
C10—C7—C8—C9	167.8 (2)	C12—N1—S1—O1	176.80 (12)
N3—C7—C10—O3	−27.4 (3)	C12—N1—S1—C1	−68.66 (15)
C8—C7—C10—O3	151.92 (17)	C2—C1—S1—O2	4.63 (13)
N3—C7—C10—C11	146.9 (2)	C6—C1—S1—O2	−177.48 (11)
C8—C7—C10—C11	−33.8 (3)	C2—C1—S1—O1	−126.05 (12)
N4—C13—C14—C15	0.5 (3)	C6—C1—S1—O1	51.84 (13)
C13—C14—C15—N5	−0.4 (3)	C2—C1—S1—N1	123.16 (13)
N5—C12—N1—S1	8.0 (2)	C6—C1—S1—N1	−58.95 (13)

### Hydrogen-bond geometry (Å, °)

**Table:**

*D*—H···*A*	*D*—H	H···*A*	*D*···*A*	*D*—H···*A*
N2—H2A···O3	0.86	2.01	2.654 (2)	131
C15—H15···O4^i^	0.93	2.46	3.262 (3)	144

Symmetry codes: (i) −*x*+1/2, *y*−3/2, −*z*+1/2.
